# Intense Co-Circulation of Non-Influenza Respiratory Viruses during the First Wave of Pandemic Influenza pH1N1/2009: A Cohort Study in Reunion Island

**DOI:** 10.1371/journal.pone.0044755

**Published:** 2012-09-12

**Authors:** Hervé Pascalis, Sarah Temmam, Magali Turpin, Olivier Rollot, Antoine Flahault, Fabrice Carrat, Xavier de Lamballerie, Patrick Gérardin, Koussay Dellagi

**Affiliations:** 1 Centre de Recherche et de Veille sur les maladies émergentes dans l’Océan Indien (CRVOI), Sainte-Clotilde, La Réunion, France; 2 Institut de Recherche pour le Développement (IRD), Sainte-Clotilde, La Réunion, France; 3 Centre d’Investigation Clinique-Epidémiologie Clinique (CIC-EC), (INSERM/CHR/Université/URMLR), Centre Hospitalier Régional, Saint-Pierre, La Réunion, France; 4 Ecologie microbienne (UMR 5557) CNRS-Université de Claude Bernard, Lyon, France; 5 Unité des Virus Emergents (UMR-S 190), IRD-Université de la Méditerranée, Marseille, France; 6 Epidémiologie des Maladies Infectieuses et Modélisation (UMR-S 707), INSERM-Université Pierre et Marie Curie, Paris, France; 7 Ecole des Hautes Etudes en Santé Publique, EHESP, Rennes, France; Faculty of Biochemistry Biophysics and Biotechnology, Jagiellonian University, Poland

## Abstract

**Objective:**

The aim of the present study was to weigh up, at the community level, the respective roles played by pandemic *Influenza* (pH1N1) virus and co-circulating human Non-*Influenza* Respiratory Viruses (NIRVs) during the first wave of the 2009 pH1N1 pandemic.

**Methods:**

A population-based prospective cohort study was conducted in Reunion Island during the austral winter 2009 (weeks 30–44) that allowed identification of 125 households with at least one member who developed symptoms of *Influenza*-like illness (ILI). Three consecutive nasal swabs were collected from each household member (443 individuals) on day 0, 3 and 8 post-ILI report and tested for pH1N1 and 15 NIRVs by RT-PCR.

**Results:**

Two successive waves of viral infections were identified: a first wave (W33–37) when pH1N1 was dominant and co-circulated with NIRVs, sharply interrupted by a second wave (W38–44), almost exclusively composed of NIRVs, mainly human *Rhinoviruses* (hRV) and *Coronaviruses (hCoV)*. Data suggest that some interference may occur between NIRVs and pH1N1 when they co-circulate within the same household, where NIRVs were more likely to infect pH1N1 negative individuals than pH1N1 positive peers (relative risk: 3.13, 95% CI: 1.80–5.46, *P*<0.001). Viral shedding was significantly shorter (P = 0.035) in patients who were co-infected by pH1N1 *and* NIRV or by two different NIRVs compared to those who were infected with only one virus, whatever this virus was (pH1N1 or NIRVs). Although intense co-circulation of NIRVs (especially hRV) likely brought pH1N1 under the detection threshold, it did not prevent spread of the pandemic *Influenza* virus within the susceptible population nor induction of an extensive herd immunity to it.

**Conclusion:**

Our results suggest that NIRV co-infections during *Influenza* epidemics may act as cofactors that contribute to shape an outbreak and modulate the attack rate. They further warrant broad spectrum studies to fully understand viral epidemics.

## Introduction

Several respiratory viruses cause *Influenza*-Like Illnesses (ILIs) in humans, including *Influenza* A (InfA) and B (InfB) viruses, *Adenoviruses* (ADV), *Respiratory Syncytial virus* (hRSV), *Enteroviruses* (EV), *Rhinoviruses* (hRV), human *Metapneumovirus* (hMPV), human *Bocavirus* (hBoV), human *Coronaviruses* (hCoV) and human *Parainfluenza* virus (hPIV) [1]–[3]. These human respiratory pathogens may express prominent seasonality and cause overlapping epidemics [4]–[6], so deciphering their dynamics requires extensive virological investigation, facilitated nowadays by molecular kits.

In April 2009, an epidemic caused by a novel triple-reassortant *Influenza* virus, pH1N1, emerged in Mexico and the United States [7] and rapidly extended worldwide causing the first *Influenza* pandemic of the 21^th^ century. Reunion Island, located in the South Western Indian Ocean, was hit by the pandemic wave during the austral winter 2009: the outbreak started on Week (W) 30 (July 20^th^), peaked on W35 (August 24^th^) and vanished on W40 (September 28^th^) [8]. The CoPanFlu-Run population-based cohort study, conducted throughout the epidemic, showed that the outbreak had impacted over 40% of the community in a strongly age-related pattern, the highest attack rates being recorded serologically in individuals less than 20 years of age [9]. Most importantly, this study revealed that almost two thirds of infections were asymptomatic or escaped medical attention.

Only few studies have investigated the full range of respiratory viruses competing for ILIs in the setting of the pH1N1 pandemic [10]–[12]. However, these studies were largely skewed towards symptomatic patients seeking medical support and thus their conclusions are hard to extrapolate for ILIs occurring in the community.

We report herein the results of a virological investigation conducted in the frame of the population-based CoPanFlu-Run cohort study. During the study period, reports of ILIs in households triggered testing for pH1N1 and NIRVs in identified individuals and all members of the same household.

Several research questions stand behind this study that are of paramount importance to the understanding of the dynamics of pH1N1 infection: i) Which NIRVs have been co-circulating with pH1N1 in the community during the course of the pandemic wave? ii) How did NIRVs spread among the different age groups before, during, and after the pandemic wave? iii) Did these viruses have significant impact on the transmission of pH1N1 between and within households? iv) Did NIRVs impact on the ultimate seroconversion rate to pH1N1?

## Methods

### Study Population

The prospective CoPanFlu-Run cohort study (772 households, 2,164 individuals) was conducted during the austral winter 2009 in Reunion Island (for details on study design, see [9]). Briefly, the inclusion phase started on July 21st (week 30) and was continued up to week 44, throughout the epidemic wave and beyond. A first serum sample (sample 1) was obtained from each household member at inclusion. An active telephonic inquiry was then conducted twice a week to record symptoms compatible with *Influenza*-like illness (ILI) occurring in households. Report of ILI (fever 37.8°C associated with any respiratory or systemic symptom) led to three consecutive visits of a nurse to the incident case-dwelling (on day 0, +3 and +8 post-report) to record symptoms and collect nasal swabs from all family members in Virocult™ tubes (∑-Virocult®, MWE). Nasal swabs were maintained at +4°C until delivery within 24 h to the laboratory. At week 45, the active inquiry was discontinued and a second (post-epidemic) serum sample (sample 2) was obtained (weeks 45–52) to determine seroconversion rates. Sera were aliquoted and stored at −80°C.

### Case Definition

ILI was defined as documented fever (≥37.8°C) with at least one symptom of Upper Respiratory Tract Infection (URTI): sore throat, cough, running and/or stopped nose, or a systemic symptom (aching). The clinical profiles of household members were assessed during the three systematic visits aimed at collecting nasal swabs and were categorized as ILIs, URTIs or asymptomatic. Familial clusters were defined as two or more members of the same household sharing the same virus. Viral co-circulation was defined as more than one virus detected in two members of the same household. A co-infection was defined as more than one virus present in the same patient, either together in the same swab, or separately in two or three consecutive swabs obtained from this individual within an 8-day.

### Detection of pH1N1/2009 and NIRVs

All nasal swab samples were spiked before nucleic acid extraction with an MS2 RNA phage as internal control [13]. qRT-PCR of MS2 RNA was run to ensure the quality of nucleic acid extraction and the absence of PCR inhibitors. Nucleic acids were extracted from 140 µL of swab supernatant using the QIAamp Viral RNA Mini Kit (Qiagen), according to the manufacturer’s protocol. Samples were subsequently screened for the presence of *Influenza* A virus RNA by qRT-PCR using a pan-*Influenza* A SYBR Green qRT-PCR assay targeting the M gene [14] (Quantitect SYBR Green qRT-PCR, Qiagen). pH1N1 detection was assessed using a pH1N1-specific TaqMan probe qRT-PCR assay targeting the HA gene (SuperScript III Platinum one-step qRT-PCR system, Invitrogen), according to the recommendations of the Pasteur Institute (Van der Werf, S. & Enouf, V., SOP/FluA/130509). A positive case was defined either as a positive qRT-PCR targeting the pH1N1 HA segment or as a positive qRT-PCR targeting the M gene followed by a confirmatory large fragment sequencing of pH1N1 for HA, NA and M segments.

The Seeplex RV15 ACE multiplex kit (Seegene) was used according to manufacturer’s recommendations to simultaneously amplify by RT-PCR, specific genomic sequences belonging to 15 human respiratory viruses. The kit detects the following viruses: InfA and InfB, hRSV-A, and hRSV-B, hRV-A,B,C, hMPV, hBoV 1,2,3,4, hCoV-229E/NL63, hCoV-OC43/HKU1, hPIV-1, hPIV-2, hPIV-3, and hPIV-4, ADV and EV [15]–[18]. An Internal Control (DNA plasmid), is included in the Seeplex RV15 ACE Detection kit to identify processed specimens containing substances that may interfere with PCR amplification. The Internal control is introduced in each amplification reaction and co-amplified with target DNA from the clinical specimen.

In addition to the 15 viral targets of the Seeplex 15 viral detection kit, described above, the Seeplex product used in the study, contained a pH1N1-specific detection system proposed by the manufacturer as a separate and unique set. At the time we have undertaken this study (i.e. 2010–2011), and as noted by others [12] the sensitivity of this additional Seeplex pH1N1 detection was rather low and we have decided not to use it. As our own detection system for pH1N1, had higher sensitivity (see below, results section), it was used throughout this study to specify pH1N1 cases according to the positive case definition reported above.

Duration of virus shedding was defined as the time between the appearance of symptoms and the last positive nasal swab. Alternatively in case of co-infection, virus shedding was directly estimated on the number of nasal swabs positive for the considered virus.

### Sequence Analysis

Positive RT-PCR products from the pH1N1 and from the other respiratory viruses were purified on a 2% gel electrophoresis using the QIAquick gel extraction kit (Qiagen), cloned in a pGEM-T Easy vector (Promega) according to the manufacturer’s instructions, and sequenced. Sequence data were analyzed using the Geneious Pro 5.3.4 software [19].

#### Serological studies

Specific antibodies to pH1N1 2009 were tittered using hemagglutination inhibition assay (HIA) as described previously [9]. Seropositivity was defined as a HIA titer of 1/40 or more as classically recommended. This HIA titer at 1/40 is considered protective, i.e. conferring 50% protection against a viral challenge [20]. In a previous study [9] we have also shown that pre-epidemic antibody titers > = 1/40 prevented seroconversion to pH1N1 and are likely protective against infection with the pandemic virus. Seroconversion to pH1N1 was defined as a shift from seronegative at inclusion (sample 1: HIA <1/40) to seropositive on follow-up (sample 2: HIA > = 1/40), or, for those sera tested seropositive on inclusion, as a four-fold increase of HIA titers between sample 1 and sample 2 paired sera.

### Statistical Analysis

Proportions were compared using Pearson Chi square test or Fisher exact test as appropriate. Age distribution was categorized in four classes (<20 yrs, 20–39 yrs, 40–59 yrs, ≥60 yrs), as done previously in the CoPanFlu-Run cohort [9]. Risk ratios (RRs) were calculated with 95% confidence intervals (95% CI). All analyses were computed in Stata (release 10; Statacorp 2008, Texas, USA). Statistical significance was set at *P* = 0.05 for all analyses.

### Ethics Statements

The prospective CoPanFlu-Run cohort study (772 households, 2,164 individuals) was conducted during the austral winter 2009 in Reunion Island in accordance to the principles expressed in the Declaration of Helsinki and French law for biomedical research (N° ID RCB AFSSAPS: 2009-A00689-48). Every eligible person for participation was asked for giving their written informed consent. We obtained informed written consent from the next of kin on the behalf of the minors/children participants involved in the study.

## Results

### Population

**Figure 1:**
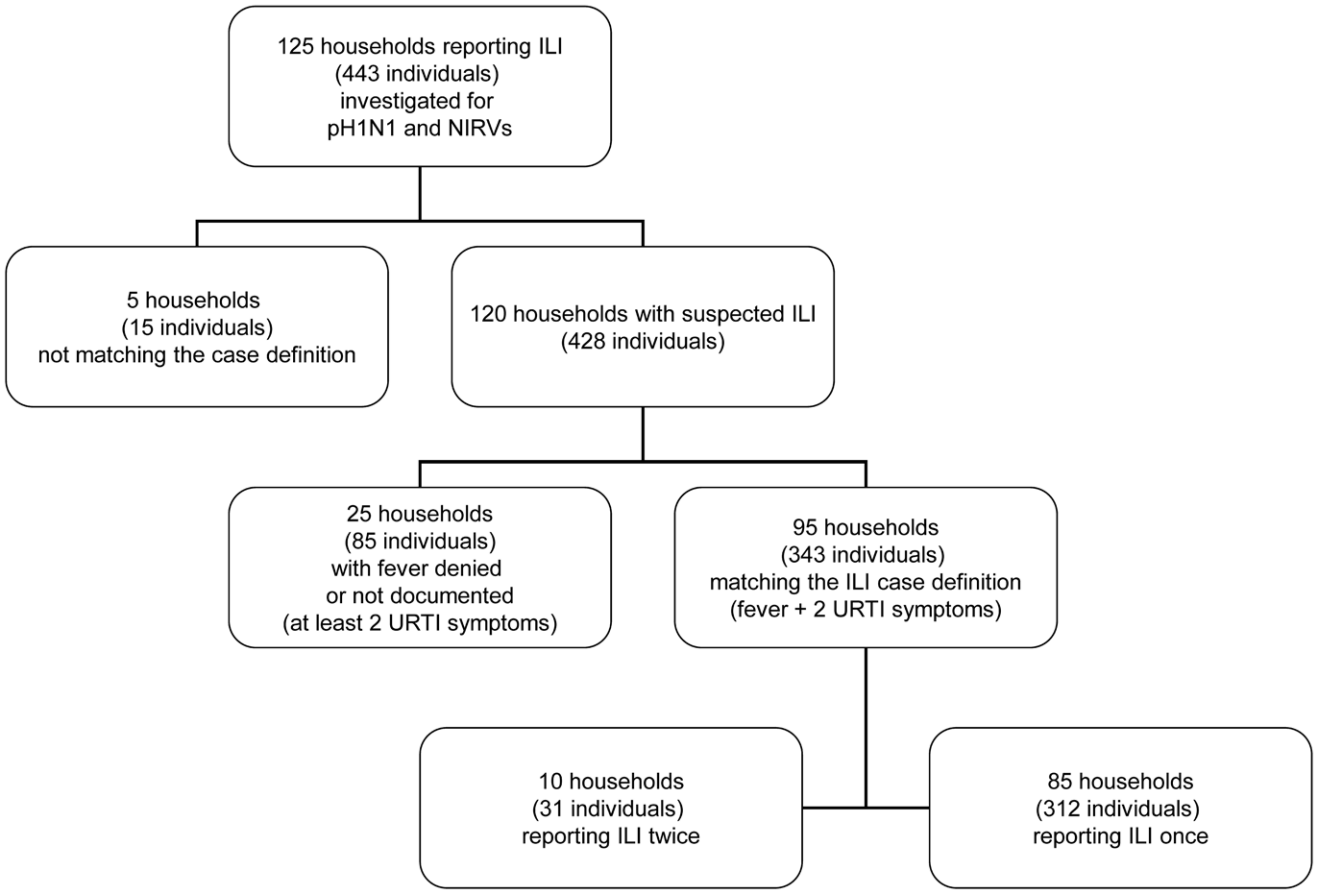
Distribution of households according to ILI case definition.

We identified 125 households (totalizing 443 individuals, M/F ratio = 0.86) with at least one member who developed ILI symptoms during the study period (W30–44). The distribution of the households according to ILI case definition is displayed in Figure 1. A posteriori review of household interview records revealed that five households did not meet the criteria of ILI or URTI (no fever and only one symptom of URTI or none). For 25 households, fever was denied or not documented, though at least one household member presented at least two symptoms of URTI (*e.g*, running nose, cough and/or sore throat). Thus, only 95 households strictly matched our initial case definition of ILI. These include ten households (31 individuals) that were visited and sampled twice because of the recurrence of ILIs a few weeks apart. Considering the large clinical spectrum of symptoms that characterize acute respiratory infections and the possibility of underreporting, all data pertaining to the 125 households were nevertheless included in the analysis. Table 1 details the demographic parameters of individuals investigated in the present study compared to the original CoPanFlu-Run cohort and to the population of Reunion Island.

**Table 1:** Distribution of age and gender within the subset of the 443 individuals tested for Non *Influenza* Respiratory Viruses (NIRVs), in the CoPanFlu-RUN cohort, and for Reunion Island community (2008 census).

	Individuals tested for NIRV^(a)^	CoPanFlu-RUN cohort^(b)^	Reunion Island population^(c)^
	N (%)	N (%)	N (%)
**AGE**			
**<20 years**	182 (41.1%)	697 (32.2%)	281,680 (35.0%)
**20–39 years**	117 (26.4%)	495 (22.9%)	224,689 (27.9%)
**40–59 years**	116 (26.2%)	614 (28.4%)	207,738 (25.8%)
**≥60 years**	28 (6.3%)	358 (16.5%)	91,393 (11.3%)
**means +/− SD**	28.7+/−19.8	36.1+/−22.5	31.7
**GENDER**			
**Male**	205 (46.3%)	1003 (46.3%)	390,645 (48.5%)
**Female**	238 (53.7%)	1161 (53.7%)	414,855 (51.5%)
**TOTAL**	443	2,164	805,500

Data are numbers and percentages, or means and standard deviations.

Age comparisons a *vs* b : *P*<0.001; a *vs* c : *P* = 0.001.

Gender comparisons a vs b : P = 0.97; a vs c : P = 0.349.

### Viro-survey

A total of 1196 nasal swabs were analyzed corresponding to 335, 88 and 20 individuals who had three (75.6%), two (19.9%) or one (4.5%) nasal swabs respectively. Considering that determinants of viral transmission between households likely differed from those within households, we analyzed the data of the viro-survey at both the household and the individual levels.

Out of the 125 households, pH1N1 virus was found 36 (28.8%) times in at least one nasal swab from at least one household member. All detected InfA viruses were of the pandemic type and no InfB virus was found. In contrast, NIRVs were frequently detected, essentially hRV, hCoV, and hPIV in 37 (29.6%), 36 (28.8%) and 18 (14.4%) households, respectively. Other viruses were less frequently found and concerned a subset of 18 households: hMPV (n = 5), ADV (n = 5), hRSV (n = 5), EV (n = 2), and hBoV (n = 1) (referred as “Other NIRVs”). The number of detected viruses was 1, 2, 3, 4 or more viruses in 55, 32, 6 and 2 households, respectively. Thirty households (24.0%) tested negative for all 16 respiratory viruses.

In order to evaluate the specificity of nucleic acid amplification by the Seeplex kit, we cloned and sequenced the amplified material identified as hRV (n = 22), hCoV (n = 20), pH1N1 (n = 15), hPIV (n = 7), hMPV (n = 7), EV (n = 1), hRSV (n = 1) and hBoV (n = 1). Sequencing and Blast analysis confirmed that each amplified sequence could be correctly ascribed to its respective virus confirming the high specificity of the Seeplex Kit [15], [18]. With regard to the sensitivity of the Seeplex kit, we found that it was lower for detection of the pH1N1 virus when compared to the qRT-PCR method: as out of the 58 positive detected by the confirmatory qRT-PCR, 10 were tested negative using the Seeplex kit.

Out of 443 individuals, 194 (43.8%) had at least one respiratory virus detected. pH1N1 was documented in 62 (14.0%), hRV in 60 individuals (13.5%), hCoV in 55 (12.4%) and hPIV in 26 (5.9%). “Other NIRVs” concerned a total of 22 individuals with the same distribution as reported in households except for hMPV which concerned 9 individuals living in 5 households. M/F sex ratios were 1.3, 1.0, 0.9, 1.3, 2.1 for pH1N1, hRV, hCoV, hPIV, and “otherNIRVs”, respectively, and 0.7 in the group of individuals tested negative to all viruses (non significant differences).

Incidences were negatively associated with age (*P*<0.001), pH1N1, hRV and “other NIRVs” being mostly detected in the youngest age group (<20 yrs): 69.4%, 61.7% and 77.3%, respectively. Age correlation was not significant for hCoV and hPIV (Table 2).

**Table 2:** Age distribution of individuals from the CoPanFlu-Run cohort tested positive for pH1N1 and NIRVs: Pandemic *Influenza* virus (pH1N1), human *Rhinovirus* (hRV), human *Coronavirus* (hCoV) or human *parainfluenza* virus (hPIV), Other NIRVs or that tested negative for viral infection despite displaying *Influenza*-like illness symptoms.

	pH1N1^(a)^	hRV^(b)^	hCoV	hPIV	Other NIRVs^(c)^	negative^(d)^
**<20 years**	43 (69.4%)	37 (61.7%)	22 (40.0%)	10 (38.5%)	17 (77.3%)	74 (29.7%)
**20–39 years**	11 (17.7%)	13 (21.7%)	17 (30.9%)	8 (30.8%)	1 (4.5%)	73 (29.3%)
**40–59 years**	7 (11.3%)	8 (13.3%)	14 (25.5%)	6 (23.0%)	2 (9.1%)	82 (32.9%)
**≥60 years**	1 (1.6%)^*^	2 (3.3%)^*^	2 (3.6%)	2 (7.7%)	2 (9.1%)^*^	20 (8.0%)
**TOTAL**	**62**	**60**	**55**	**26**	**22**	**249**

Data are numbers and percentages.

b *vs* a : *P* = 0.827; b *vs* f : *P*<0.001.

c *vs* a : *P* = 0.010; c *vs* f :*P* = 0.351.

d *vs* a : *P* = 0.031; d *vs* f :*P* = 0.699.

e *vs* a : *P* = 0.206; e *vs* f : *P<*0.001.

Statistical significance was set at P = 0.05 for all analyses, except for Table 2 for which a correction of Bonferroni was applied at P = 0.0125 to account for multiple comparisons.

Familial clusters (i.e. two or more members of the same household sharing the same virus), were observed in 13 households out of 36 (36.1%) for pH1N1, in 14/37 households (37.8%) for hRV, in 12/36 households (33.3%) for hCoV, in 5/18 households (27.8%) for hPIV and in 3/18 (16.7%) for “other NIRVs”, respectively (Chi square test 4 ddl: 2.95, *P* = 0.566).

We also checked whether pH1N1 and NIRVs could mutually interfere for the occurrence of familial clusters. No statistically significant differences could be demonstrated (data not shown).

### Temporal Dynamic of pH1N1 and NIRVs Infections

**Figure 2:**
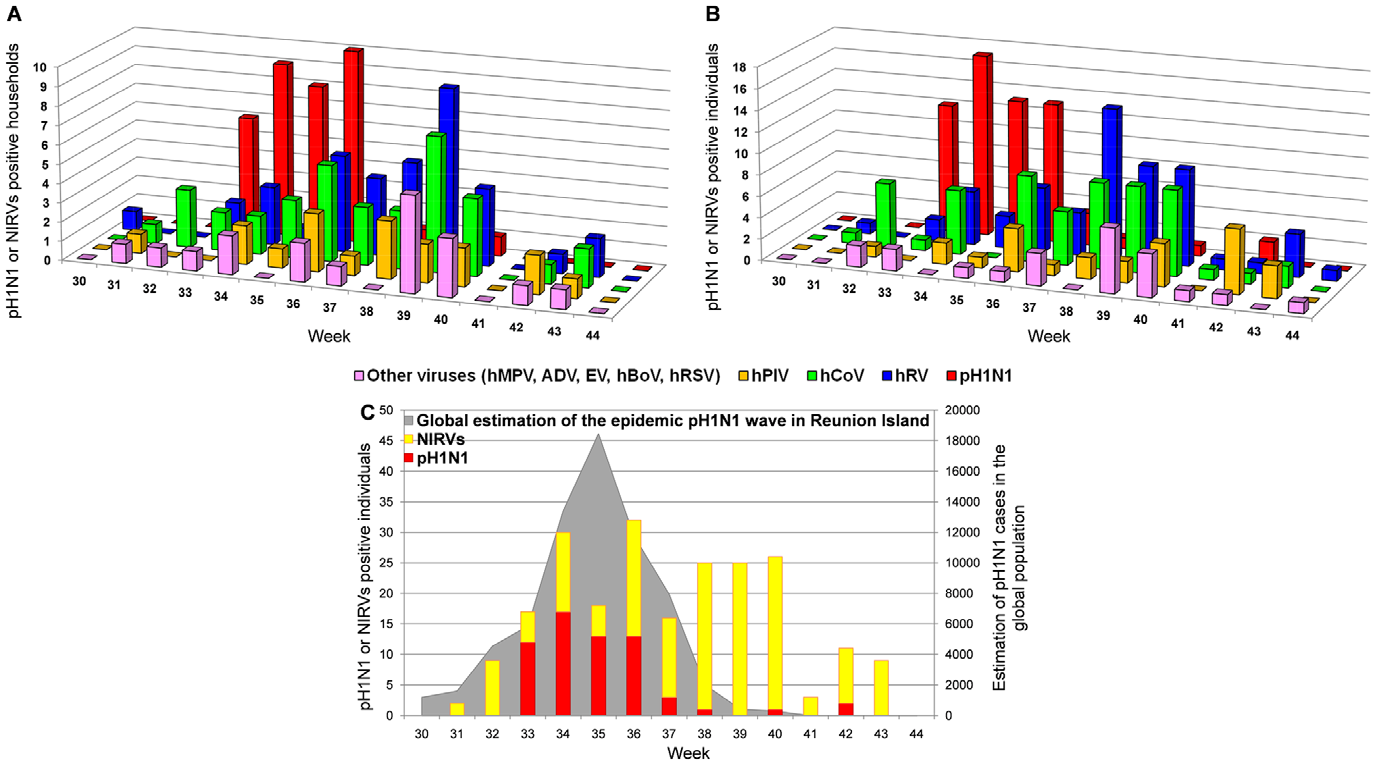
Temporal dynamics of pH1N1 and NIRVs between weeks 30 and 44 of 2009. Charts depict the total number of households (**A**, n = 125) or individuals (**B**, n = 443) that tested positive for pandemic *Influenza* (pH1N1, red), *human Rhinovirus* (hRV, blue), *human Coronavirus* (hCoV, green), *human parainfluenza virus* (hPIV, orange), other viruses (pink). **C** depicts the number of positive pH1N1 or NIRV cases in this cohort with respect to the estimated global impact of the pH1N1 epidemic (shaded area), as reported by the Public Health Department of Reunion Island [8].

The temporal dynamic of pH1N1 and NIRVs is shown in Figure 2A for the 125 households and in Figure 2B for the 443 individuals. The passage of the pandemic pH1N1 wave in Reunion Island was very sharp. In fact, the epidemic upsurge only lasted five weeks (W33–37) during which almost all households (34/36) and all individuals (60/62) positive for pH1N1 were detected. A total of 58 households and 228 individuals were sampled during this epidemic window. NIRVs were largely circulating and detected in 43 households during this short period (hRV and hCoV in 15, hPIV in 7 and “other NIRVs” in 6, respectively). For individuals, hRV and hCoV were detected in 20, hPIV in eight and other NIRVs in seven persons. Overall, two successive waves of viral infections were identified: a first wave (W33–37) when pH1N1 co-circulated with various NIRVs, followed by a second wave that peaked on W38, composed almost exclusively of NIRVs, mainly hRV and hCoV. The temporal dynamics of the detected viruses within individuals was similar to that observed within households for pH1N1 and hRV infection, but not for hCoV which was more evenly distributed along the whole observation period. Figure 2C is a synoptic view of positive virus isolation compared to the pH1N1 epidemic wave as reported by the Public Health Department of Reunion Island. Considering that true co-circulation of pH1N1 and NIRVs was restricted to W33–37, data analysis was focused on this specific period in the following sections.

### Co-circulation and Co-infections by pH1N1 and NIRVs

During W33–37, at least one different NIRV co-circulated with pH1N1 in 21 households out of the 34 where the pandemic virus was detected (hRV, hCoV, hPIV, and “other NIRVs”, in 8, 8, 3 and 2 households respectively). Data were stratified according to the presence or absence of pH1N1 virus and analyzed for the presence or absence of NIRVs. Similarly, data were ranked according to the detection of hRV, hCoV or hPIV in the nasal swabs at day 0 and analyzed for the presence or absence of pH1N1 during the eight days of nasal sampling. Despite the fact that pH1N1 appeared to more readily infect households where hRV did not circulate (and *vice versa*), differences did not reach statistical significance (data not shown).

Out of the 194 individuals who were detected positive for at least one respiratory virus (pH1N1 or NIRV), 31 individuals had evidence of viral co-infection (Table 3). Co-infecting viruses were detected in the same nasal swab for 18 individuals and in successive swabs for 13 individuals (8-day period). Duplex co-infections concerned 29 individuals, and multiple (triple) co-infections two individuals. Co-infections concerned two and three viruses in 29 and two individuals, respectively. Co-infection included pH1N1 for only eleven individuals, associated with hRV in four, hCoV in five and hPIV in two. In the 21 households in which more than one virus were detected, NIRVs were significantly more likely to infect pH1N1 negative individuals than pH1N1 positive peers (71.7% *vs* 22.9%, RR: 3.13, 95% CI: 1.80–5.46, *P*<0.001).

**Table 3:** Matrix of viral co-infections.

	pH1N1	hRV	hCoV	hPIV	ADV	EV	hRSV	hMPV	hBoV
**pH1N1**	–	1	3	1	0	0	0	0	0
**hRV**	3	–	3	1	1	0	0	1	0
**hCoV**	2	1	–	1	0	1	0	0	0
**hPIV**	1	2	2	1	0	0	0	0	0
**ADV**	0	2	1	0	–	0	0	0	0
**EV**	0	1	0	0	0	–	0	0	0
**hRSV**	0	0	1	0	0	0	–	0	0
**hMPV**	0	0	0	0	0	0	0	–	0
**hBoV**	0	1	0	0	0	0	0	0	–

Number of individuals co-infected with combinations of different viruses: Above the diagonal; individuals that tested positive for more than one virus in consecutive swabs (8 days apart). Below the diagonal; individuals that tested positive for more than one virus in the same swab.

### Duration of pH1N1 and NIRVs Shedding

Of 335 individuals for whom three nasal swabs were obtained on day 0, 3 and 8, the mean duration (+/−SD) of viral shedding was 7.6 (+/−3) days for pH1N1, 10.4 (+/−5.1) days for hRV, 7.8 (+/−3.8) days for hCoV and 9.3 (+/−3.2) days for hPIV. We then estimated the duration of viral shedding for pH1N1-NIRVs co-infections among the samples detected positive for any virus. Out of eleven individuals positive for pH1N1 that had evidence of co-infection, the pH1N1 virus was detected in one single swab for seven individuals, in two swabs for two, and in three swabs for two. With regard to viral shedding of co-infecting NIRVs, they were detected in one single swab in ten cases, and in three swabs in one case. When pooled together, these data indicate that the duration of pH1N1 and NIRVs viral shedding was significantly shorter in patients with co-infection compared to those with single infection (data not shown). Hence, viral positivity restricted to only one single swab was found in 16/20 (80.0%) episodes of viral co-infection but in only 87/157 (55.4%) episodes of single viral infection (Chi square test 1 ddl: 4.40, *P* = 0.035).

### Clinico-biological Patterns

The clinical picture of 367 individuals for whom data were available was correlated to virus detection at day 0, after exclusion of co-infections. Almost ninety percent (35/39) of individuals with RT-PCR documented pH1N1 infection were symptomatic (i.e. either ILI or URTI) and most of them (31/39) had fever. Patients with NIRVs reported fever less frequently (26/62) compared to those with pH1N1 infection (41.9% vs. 79.5%; RR: 0.56, 95%CI: 0.14−0.76, *P*<0.001). Nearly one third of patients with NIRVs presented URTI (20/62) and one quarter (16/62) had no symptoms.

During the epidemic upsurge (W33–37) almost half of patients with ILIs were pH1N1 positive, 16.5% had documented NIRVs and 35.3% were negative. Of these three categories, the proportions of patients that reported URTIs were 19.5%, 26.2% and 54.8% respectively (Table 4). Almost half of the 114 individuals who tested negative were yet symptomatic for ILI (26.3% = 30/114) or URTI (20.2% = 23/114), we believe reflecting either some lack of sensitivity for viral detection in our study, or infection by other pathogens.

**Table 4:** Correlation between clinical symptoms at presentation and PCR detected viruses.

	pH1N1^(a)^	hRV^(b)^	hCoV^(c)^	hPIV^(d)^	Other NIRVs^(e)^	Negative swab^(f)^
**Weeks 30–32 ^(T0)^**						
**ILI (N = 5)**	0 (0.0%)	0 (0.0%)	2 (40.0%)	0 (0.0%)	0 (0.0%)	3 (60.0%)
**URTI (N = 3)**	0 (0.0%)	0 (0.0%)	2 (66.7%)	0 (0.0%)	0 (0.0%)	1 (33.3%)
**Asymptomatic** **(N = 10)**	0 (0.0%)	1 (10.0%)	1 (10.0%)	0 (0.0%)	1 (10.0%)	7 (70.0%)
**Weeks 33–37 ^(T1)^**						
**ILI (N = 85)**	41 (48.2%)	7 (8.2%)	4 (4.7%)	2 (2.4%)	1 (1.2%)	30 (35.3%)
**URTI (N = 42)**	8 (19.0%)	5 (11.9%)	5 (11.9%)	1 (2.4%)	0 (0.0%)	23 (54.8%)
**Asymptomatic** **(N = 70)**	1 (1.4%)	3 (4.3%)	3 (4.3%)	0 (0.0%)	2 (2.9%)	61 (87.14%)
**Weeks 38–44 ^(T2)^**						
**ILI (N = 56)**	2 (3.6%)	13 (23.2%)	6 (10.7%)	5 (8.9%)	3 (5.4%)	27 (48.2%)
**URTI (N = 55)**	0 (0.0%)	8 (14.6%)	9 (16.4%)	3 (5.4%)	3 (5.4%)	32 (58.2%)
**Asymptomatic (N = 85)**	0 (0.0%)	6 (7.1%)	5 (5.9%)	6 (7.1%)	2 (2.4%)	66 (77.5%)

Number of individuals (percentages) with pandemic *Influenza* (pH1N1), human *rhinovirus* (hRV), human *Coronavirus* (hCoV), human *parainfluenza* virus (hPIV), other Non *Influenza* Respiratory Virus (NIRVs); or negatively-testing individuals that presented with symptoms of *Influenza*-like illness (ILIs), Upper Respiratory Tract Infections (URTIs) or were asymptomatic.

Data correspond to individuals included in the cohort during weeks 30–32 (T0), weeks 33 to 37 (T1) or weeks 38–44 (T3).

Comparisons of clinical expression between T1 and T2 for each virus: pH1N1: *P*<0.001; hRV : *P* = 0.006; hCoV : *P* = 0.038; hPIV : *P* = 0.001; Others NIRVs : *P* = 0.001; Negative swabs : *P* = 0.016.

Seroconversion rates were deduced from antibody titration to pH1N1 on paired blood samples collected from each individual. For the present study, we considered only the 170 pairs of sera for which the first blood sample was collected during the transmission window (W33–37) and the second at the end of the study, after W45. We correlated the seroconversion rates of these pairs to results of the viro-survey. Interestingly, the seroconversion rate to pH1N1 was 67.6% (23/34) for those tested pH1N1+/NIRVs–; 39.2% (38/97) for those tested pH1N1−/NIRVs–; 46.7% (14/30) for those tested pH1N1−/NIRVs+ and 77.8% (7/9) for those tested pH1N1+/NIRVs+. Hence, serologic data indicate that the pandemic virus has largely spread among the study population during the epidemic upsurge and had induced protective immunity, regardless of the infecting virus detected by molecular amplification.

## Discussion

Our viro-survey confirmed the very fast passage of the pandemic *Influenza* virus through the Reunion Island community: the epidemic wave did not last more than 5 weeks (W33–37) during which almost all of households and individuals tested positive for pH1N1 were clustered. Interestingly, the pandemic *Influenza* virus completely prevented the expression of any seasonal *Influenza* types A (H3N2) and B viruses despite the fact they were detected earlier in the season [21]. This strongly contrasts with the intense observed circulation of NIRVs during the whole observation period (W30–44), when at least one NIRV (mainly hRV, hCoV and hPIV) was detected in 87.2% of tested households and in 37.0% of tested individuals and pH1N1 was detected in 28.8% of tested households and 14.0% of tested individuals. This also holds true even during the peak of the pH1N1 epidemic (W33–37), when almost as many NIRVs as pH1N1 were documented. If one consider the short duration of pH1N1 transmission and its abrupt interruption on week 37, the intense viral co-circulation may suggest some reciprocal interactions between these respiratory pathogens and hence a possible role for NIRVs in shaping the *Influenza* epidemic.

The highest rate of pH1N1 detection was recorded in young individuals, confirming our previous observations based on serologic data [9]. Similar age-related patterns were observed for hRV and “other NIRVs” (hMPV, ADV, hRSV, EV and hBoV), whereas hCoV and hPIV were more evenly distributed over all age groups. The scarcity of RSV and hMPV in our series likely reflects the strong seasonality of these pathogens which depends on geographic location and altitude [22], [23]. RSV was reported to occur in Reunion Island [24] and in Madagascar [25], mainly during the austral summer (the hot and humid season) and not during winter which is the season window of our study. In addition RSV occurs primarily in infants less than 2 years old and this age group was represented in our series by only 23 individuals. A study in Antananarivo (Madagascar) has also detected only few hMPV from patients with ILI [25].

Familial clustering of pH1N1 or hRV or hCoV was observed in almost one third of positive households. Most interestingly, NIRVs were not uniformly distributed over time as they were most commonly detected just after the passage of the *Influenza* pandemic wave, with hRV forming a second epidemic wave that peaked at week 38.

Viral co-infection during W33–37 concerned less than 5% of investigated individuals, a low incidence which has been similarly observed in other studies [26]. In contrast, a much higher rate of NIRVs and pH1N1 co-circulation (27.6%) was observed within households.

pH1N1 and NIRVs tended to be mutually exclusive when co-circulating together within the same household: in such cases, NIRVs were more likely to infect pH1N1 negative individuals than pH1N1 positive peers (RR: 3.13). Similar trends were also observed with hRV and hPIV, though they did not reach statistical significance, possibly due to the small sample size.

The mean total duration of pH1N1 viral shedding in our population-based cohort was estimated at 7.6 (+/−3) days for pH1N1, a figure consistent with those reported in the literature among hospitalized or pediatric patients [27]–[30].

Interestingly, when data recorded for pH1N1 and NIRVs were pooled together regardless of the specific virus, the duration of viral shedding was significantly shorter (*P* = 0.035) in the context of viral co-infection compared to single infection, a further argument suggesting viral interference during co-infection.

This study has outlined various features of the dynamics of viral co-circulation during the studied epidemic: i) pH1N1 and NIRVs (especially hRV), though largely overlapping, essentially spread during two successive waves; a pH1N1 wave followed by a hRV wave that peaked on W34 and W38, respectively. ii) During W33–37, when pH1N1 and NIRV waves overlapped, the global trend was to infect different households separately. iii) In households where pH1N1 and NIRVs co-circulated, co-infection was less likely than mutually exclusive infections of different individuals by the different viruses. iv) Viral co-infection in the same individual was rare and when it occurred, excretion of either of the co-infecting virus tended to be shorter.

All these features suggest a negative interplay between NIRVs (especially hRV) and pH1N1, and suggest that competition may have played a role in the extinction of pandemic *Influenza* virus transmission on W38. Similar observations based on epidemiologic data have been previously reported [31]–[33]. The mechanism of interplay is likely to be of immune origin: the first virus infecting one individual may activate the innate immunity (particularly INF α/β pathways), hindering the invasion of a second virus. However, two facts suggest that this interplay is likely only partial: i) the highest seroconversion rate to pH1N1 was observed in the youngest age group which is also the main target of co-circulating hRV [26]; ii) the seroconversion rate to pH1N1 of individuals who tested RT-PCR negative to pH1N1 during W33–37 was high and not significantly different from that recorded in individuals who have been tested RT-PCR positive for NIRVs only. As pH1N1 transmission took place almost exclusively during W33–37 (i.e. it was almost totally interrupted after W37), the seroconversion to pH1N1 attested on pairs of sera in which the first blood sample was obtained during W33–37, indicates viral infection taking place during this short period. Thus, the high seroconversion rates to pH1N1 that we observed during this very period, attests regardless of the qRT-PCR status of the individuals, the large diffusion of the pandemic virus within the study population. Hence, the intense co-circulation of NIRVs (and especially hRV) with pH1N1 did not apparently hinder the silent spread of the pandemic virus in the exposed population, nor did it prevent the induction of a solid herd immunity which in turn would likely have participated in interrupting the pH1N1 transmission. If such an assumption was correct, then the intense co-circulation of NIRVs and pH1N1 during the pandemic wave may account for the overall benign nature of the pH1N1-related pathologies in Reunion Island, by reducing the pH1N1 burden and shortening the duration of the epidemic through viral competition.

In our study, individuals who were positive for pH1N1 virus were also mostly symptomatic and presented the classic clinical picture of ILI, including the presence of fever. Reciprocally, symptomatic individuals (i.e. presenting with either ILI or URTI) during the upsurge of epidemic (W33–37) were virologically documented three times more as pH1N1 positive than as NIRVs positive. This information may help to assess the real burden of pH1N1 during the 2009 pandemic.

Co-circulation of *Influenza* virus and NIRVs represents an interesting example of community pathocenosis [34]. This concept highlights the complex interplays that are often observed when multiple pathogens infect a single host and impact the host response to each pathogen. This may alter disease severity and change the clinical picture induced by the co-infecting agent. For instance herpesvirus latency confers resistance against superinfection by a lethal pathogen: mice latently infected with either murine gamma herpesvirus 68 or murine cytomegalovirus, become resistant to infection with the lethal bacterial pathogens Listeria monocytogenes and Yersinia pestis [35]. In humans, co-infecting pathogens activate HIV replication and accelerate disease evolution due to the retrovirus [36]. Reciprocally, HIV infection impacts on disease prevalence and disease transmission in tuberculosis and malaria and many other co-incident diseases [37], [38]. It was suggested that this interplay may be driven by alterations in cytokine balance induced by co-pathogens [35], [39].

Acting as viral cofactors, NIRVs may vary in dominant viral types and frequency from one country/region to another [40] according to local epidemiological conditions, seasonality, temperature and humidity and may account together with other individual factors (i.e. pre-existing immunity and infectious background) for the large diversity in terms of epidemic intensity and severity that was reported at the global level with the pH1N1 pandemic [41].

Our results, obtained through a prospective study conducted at the community level, confirm previous conclusions from reports of hospitalized patients [42]. They lend support for investigating *Influenza* virus epidemics in perspective broader context that takes into consideration local epidemiological specificities including co-circulating viral respiratory pathogens that have here been highlighted as possible cofactors, influencing the shape of epidemics and modulating viral attack rates.
